# HEALTH CARE COSTS OF MEDICARE BENEFICIARIES WITH FRAILTY AND/OR DEMENTIA

**DOI:** 10.1093/geroni/igae098.2454

**Published:** 2024-12-31

**Authors:** Stephanie Denise Sison, Sandra Shi, Gahee Oh, Brianne Olivieri-Mui, Chan Mi Park, Ellen McCarthy, Dae Hyun Kim

**Affiliations:** University of Massachusetts Chan Medical School, Worcester, Massachusetts, United States; Harvard Medical School/Hebrew SeniorLife, Boston, Massachusetts, United States; Hebrew SeniorLife, Boston, Massachusetts, United States; Northeastern University, Boston, Massachusetts, United States; Hebrew SeniorLife, Boston, Massachusetts, United States; Harvard Medical School, Boston, Massachusetts, United States; Hebrew SeniorLife, Boston, Massachusetts, United States

## Abstract

Frailty and dementia predispose older adults to adverse health outcomes, resulting in increased health care utilization. In this study, we examined healthcare costs in a 5% sample of Medicare fee-for-service (FFS) beneficiaries using Medicare part A and B claims from 2016-2019. Using validated claims-based algorithms, we identified the presence of dementia and frailty (claims-based frailty index ≥0.25) in community-dwelling beneficiaries, 65 years or older, with continuous FFS enrollment 12 months prior to the start of each calendar year. Annual costs per beneficiary were calculated across 4 groups: a) those having dementia alone, b) frailty alone, c) both frailty and dementia, and d) neither. Having frailty alone incurred the highest total annual cost ($38,119), followed by frailty and dementia ($33,098) and then dementia alone ($16,622). The breakdown of costs revealed that inpatient costs (highest proportion of total costs) and carrier costs (e.g., provider fees, labs, imaging, procedures; second-highest proportion of total costs), followed a similar pattern as the total costs. This pattern persisted when examining preventable inpatient costs, with the frailty alone group having the highest costs ($2,517), followed by the frailty and dementia group ($1,610). SNF costs were highest in the frailty and dementia group ($5,338), followed by the frailty only group ($4,044), and then the dementia only group ($2,208). Our findings suggest that the presence of frailty, regardless of dementia status, drives healthcare cost. This highlights the need for a holistic patient-centered approach to optimize and reduce costs for frail older adults.

